# One-Dimensional Molecular Coordinate Orders Amino Acids across Water-Mediated Processes

**DOI:** 10.1021/acs.jcim.6c01897

**Published:** 2026-08-08

**Authors:** Yusung Ok, Youngjune Park

**Affiliations:** † Department of Environment and Energy Engineering, Gwangju Institute of Science and Technology (GIST), 123 Cheomdangwagi-ro, Buk-gu, Gwangju 61005, Republic of Korea; ‡ Research Center for Innovative Energy and Carbon Optimized Synthesis for Chemicals (Inn-ECOSysChem), Gwangju Institute of Science and Technology (GIST), 123 Cheomdangwagi-ro, Buk-gu, Gwangju 61005, Republic of Korea

## Abstract

Amino acid behavior in water-mediated processes is not well captured by hydrophobicity or side-chain class alone, because residues with similar hydropathy can differ in charge localization, geometry, surface exposure, and hydration response. The objective of this study is to determine whether integrating complementary molecular representations can reveal a chemically interpretable molecular coordinate that organizes amino acid behavior across distinct water-mediated processes. We fused electronic-, structural-, and solvation-level representations into a similarity network and embedded it spectrally to obtain a one-dimensional coordinate, *Z*_F_, which captures coupled variation in electrostatics-, geometry-, and hydration-related features. Across gas hydrate formation, ice recrystallization inhibition, and CaCO_3_ crystallization, *Z*_F_ provides a consistent residue-level coordinate and captures interaction trends that differ in direction from those described by the Kyte–Doolittle hydropathy scale in the latter two systems.

## Graphical abstract

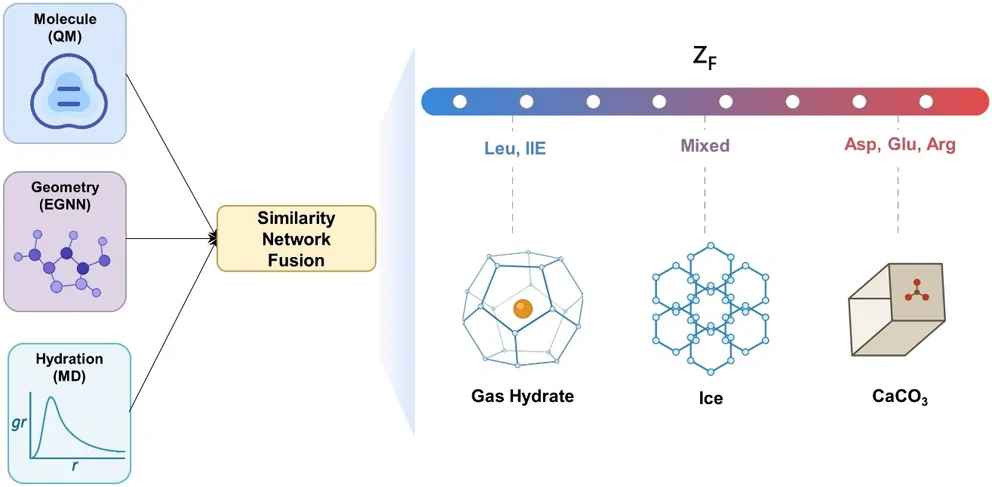

## Introduction

Amino acids provide a compact set of molecular building blocks for examining how small structural differences shape aqueous interactions. Their chemical diversity makes them useful as biofriendly additives and as model compounds for more complex peptides and proteins.^1–3^ However, their behavior in water is not determined solely by side-chain class. One-dimensional descriptors such as hydrophobicity or formal charge compress information about charge localization, side-chain geometry, surface exposure, and hydration response into a single scalar, and so they capture only part of the hydration-related molecular organization when several interaction modes act together. Among the established hydropathy scales, the Kyte–Doolittle (KD) scale is grounded in side-chain transfer between bulk aqueous and nonpolar environments and therefore matches the bulk-aqueous setting of water-mediated processes.^4^

This challenge is not specific to hydrophobicity. Each major descriptor family captures one facet of the relevant chemistry: electrostatic representations encode charge heterogeneity and local polarity; graph-level representations encode connectivity and functional group arrangement; and solvation-level descriptors capture surface exposure and hydration shell organization.^5^ Used separately, these views can yield partial or inconsistent molecular organizations. A useful coordinate for amino acid modulation should integrate these complementary signals without allowing any single descriptor family to dominate the molecular landscape.

We address this challenge with a multiview unsupervised framework that combines electronic-, structural-, and solvation-level representations into a molecular similarity network. Similarity network fusion (SNF) integrates view-specific neighborhood structures into a consensus graph,^6^ complementing alternative multiview integration strategies based on regularized multiple kernel learning,^7^ joint clustering across modalities,^8^ and probabilistic factor analysis.^9^ From this fused landscape, a one-dimensional coordinate, *Z*_F_, is extracted that arranges residues according to coupled variation in electrostatics, molecular geometry, and hydration structure.

Gas hydrate formation, ice recrystallization inhibition (IRI), and calcium carbonate (CaCO_3_) crystallization serve as three test beds for water-mediated ordering. Gas hydrates provide the initial case because promotion-like and inhibition-like behaviors occur within the same amino acid library.^1^ The hydrate-derived organization separates low-*Z*_F_ nonpolar residues, mixed-character residues, π/sulfur-motif residues, and high-*Z*_F_ polar or charge-segmented residues. Projection onto independent IRI and CaCO_3_ crystallization data tests whether the hydration-related molecular organization captured by the multiview-derived coordinate retains chemical meaning across distinct water-mediated processes.^2,3^

To address this gap, the present study is motivated by the hypothesis that water-mediated molecular organization cannot be adequately described by conventional one-dimensional molecular interaction dimensions, because it arises from the coupled effects of molecular geometry, electrostatics, and hydration structure. The central research question is whether integrating complementary molecular representations through a multiview similarity network can identify a chemically interpretable coordinate.

## Results and Discussion

### A Multiview Representation Reveals the Property-Based Organization of Amino Acids

To characterize the chemical factors that distinguish amino acids in aqueous environments, three complementary molecular views were constructed, each capturing a different interaction pathway (Figure 1a). Feature set A describes the electronic and geometric landscape of each residue, encoding the spatial charge distribution, local electrostatic contrast, surface patch organization, and global shape. Feature set B, a graph-level representation, summarizes the underlying molecular scaffold through connectivity and functional group arrangement, providing an orientation-independent account of the chemical topology. Feature set C characterizes solvation-level behavior, quantifying surface exposure, the nonpolar–polar balance, and hydration-accessible geometry under equilibrium aqueous solvation conditions.

**Figure 1:**
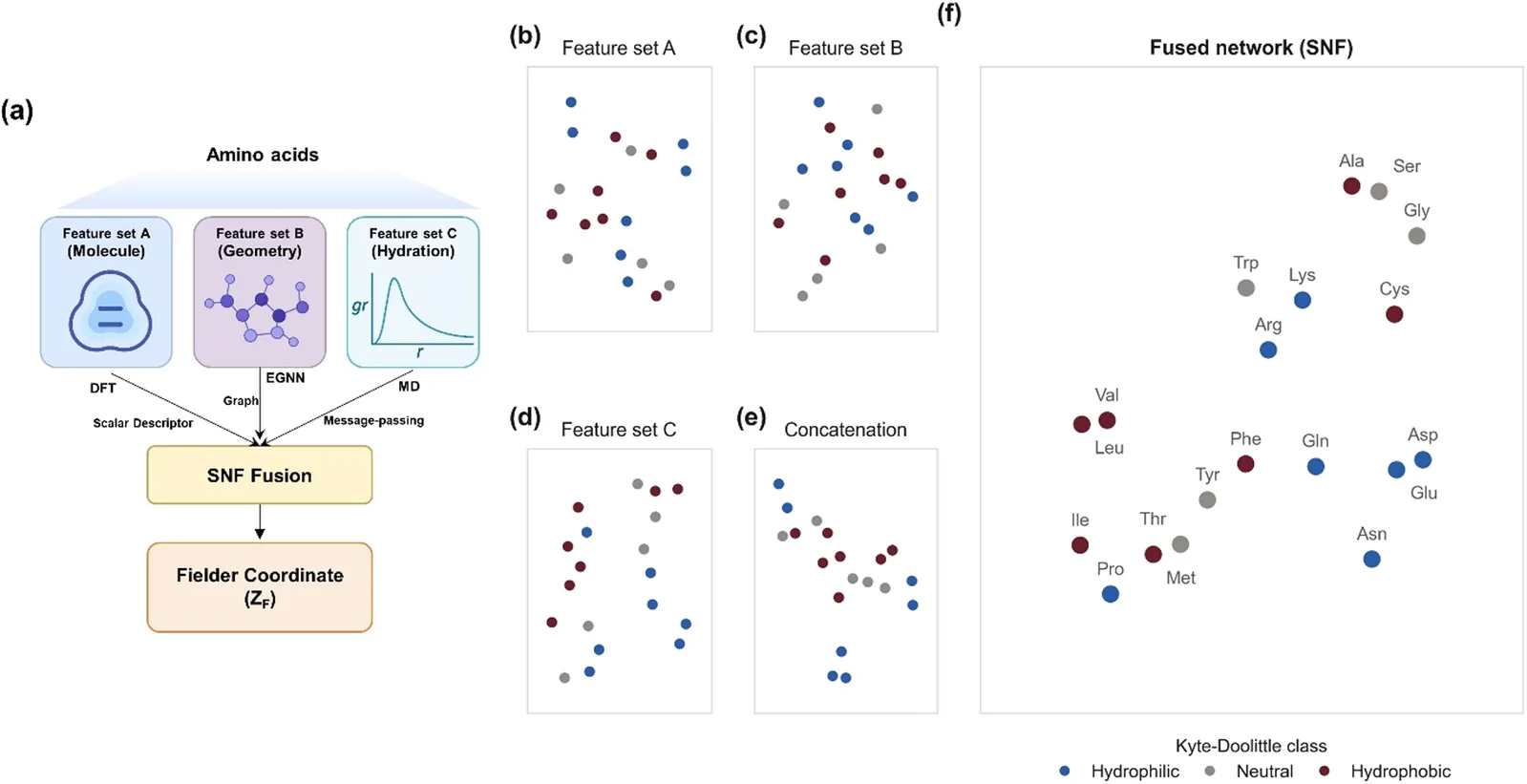
Multiview similarity fusion integrates complementary molecular representations into a unified similarity landscape. (a) Electronic structure (Feature set A), molecular geometry (Feature set B), and hydration representations (Feature set C) are integrated using similarity network fusion (SNF) to generate the fused molecular coordinate, *Z*_F_. (b–d) t-SNE projections of individual feature set representations. (e) t-SNE projection of the naive concatenation of all descriptors, showing dominance by Feature set A. (f) t-SNE projection of the SNF-derived fused similarity network. Points are colored according to Kyte–Doolittle (KD) hydropathy classes. Unlike descriptor concatenation, which reproduces Feature set A distances (99.9% of Euclidean variance; ρ = 1.000), SNF integrates complementary neighborhood information across representations.

These three perspectives provide complementary information on electrostatics, topology, and solvation structure, and together capture the molecular interaction factors relevant to aqueous modulation. Histidine was excluded because its protonation and tautomeric variability preclude a consistent single-form representation. Quality-control analyses for feature set A patch segmentation, feature set B embedding training, and feature set C molecular dynamics (MD) equilibration are presented in the Supporting Information, Figures S1–S3. A stepwise overview of the full multiview unsupervised pipeline is presented in the Supporting Information, Note S1.

Each descriptor family, embedded on its own, captures only part of the molecular landscape (Figure 1b–d).^10^ The electronic descriptors (Figure 1b) resolve polarity and charge contrast but conflate geometrically distinct molecules. Graph-level embeddings (Figure 1c) capture connectivity patterns yet lack the resolution to form well-defined groupings. The solvation descriptors (Figure 1d) exhibit the strongest global gradient along a hydrophobic–hydrophilic axis, but blur the distinctions among polar and mixed-character residues. Simply concatenating the three feature families (Figure 1e) does not provide an effective integration of the complementary molecular information. The resulting representation is dominated by Feature set A, particularly the surface area and ESP variance descriptors, which account for 99.9% of the total variance. The pairwise-distance matrix of the concatenated space is indistinguishable from that of Feature set A alone (ρ = 1.000), indicating that the contributions of Feature sets B and C are largely obscured. This behavior originates from differences in descriptor scale rather than from differences in the information content of each representation. Therefore, the three feature families were integrated using SNF, which places each representation on a common similarity scale and enables their combined contribution to the molecular landscape.

To integrate these complementary signals, similarity network fusion (SNF)^6^ combines the three feature set similarity networks. Embedding the fused network yields a markedly clearer molecular organization than any single-feature set embedding or native feature concatenation (Figure 1f). Although feature set C contributes strongly to the global gradient, the fused coordinate incorporates additional neighborhood refinements from electronic and topological representations that are absent from the individual solvation landscape. These additional signals refine the neighborhood relations and yield a consensus landscape.

In the fused space, hydrophobic residues formed a tight cluster with polar and charged residues positioned well away from them. Mixed-character residues, whose positions had varied across the individual feature sets, now lay between the two groups. The structure remained stable under perturbations to the input feature sets, indicating consistent cross-view reinforcement, even though the contributions are not perfectly symmetric. Residues also lay along smooth gradients, indicating that continuous directions of variation are already encoded in the landscape. SNF convergence and prediffusion fused similarity consistency are quantified in Supporting Information, Figure S4. The fused similarity network yielded a low-dimensional latent landscape that captured the shared structure across the electrostatic, geometric, and solvation feature sets. Amino acids formed neighborhoods along a curved, elongated geometry with a leading direction of continuous variation. The principal direction aligned with this geometry defines the modulation coordinate, *Z*_F_, which is a one-dimensional axis that summarizes the multiview representation while preserving residue-level relationships.

To show what each feature set encodes, a representative residue, asparagine, is rendered under the three representations (Figure 2a–c): DFT-optimized structure, the graph consumed by the equivariant network, and its first hydration shell from a molecular dynamics frame. The modulation coordinate *Z*_F_ is the leading spectral direction of the fused network, defined as the Fiedler vector of the random-walk graph Laplacian.

**Figure 2:**
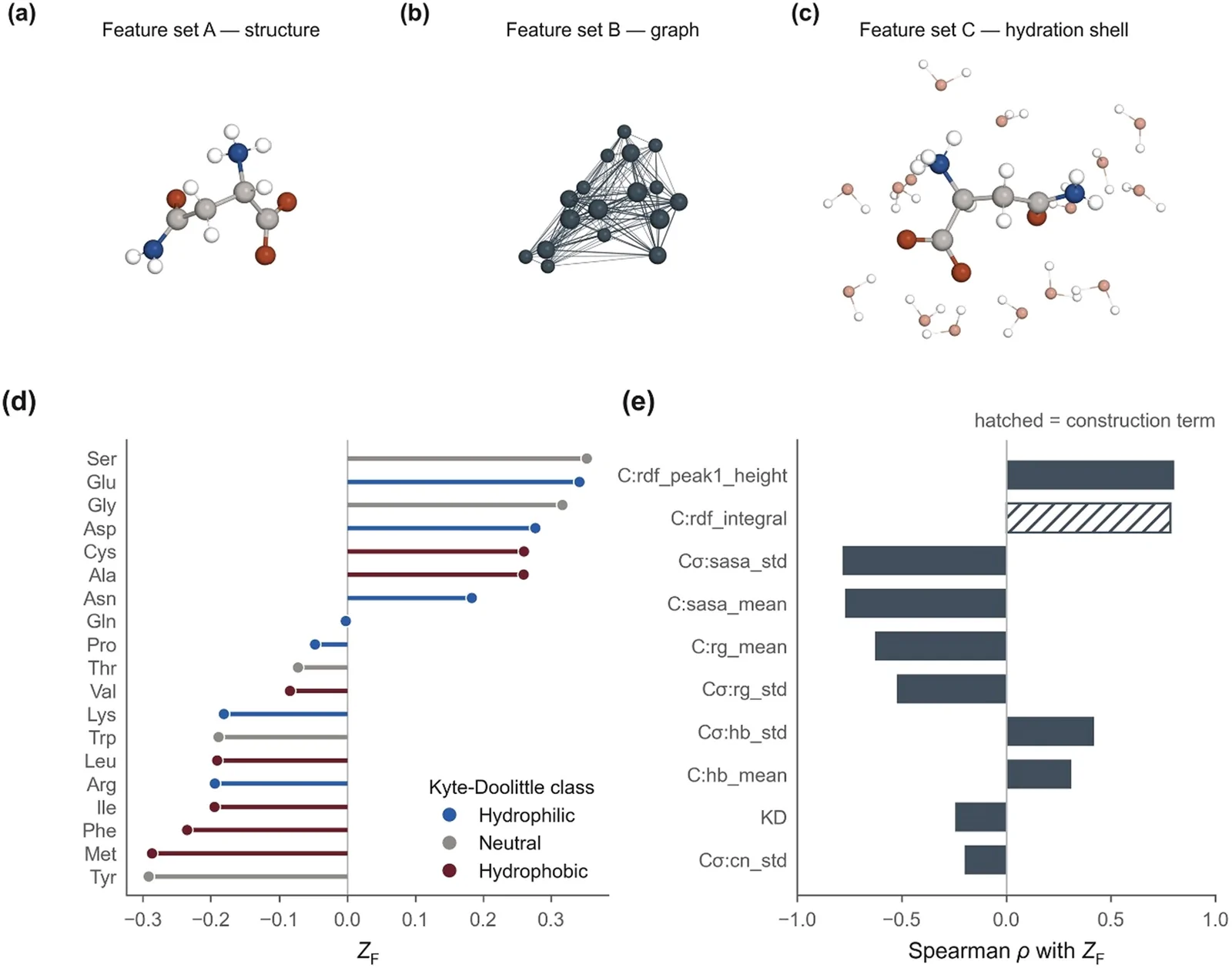
Molecular interpretation of the fused coordinate through structural and hydration representations. (a–c) Asparagine represented by the three feature sets: (a) DFT-optimized structure, (b) EGNN input graph, and (c) first hydration shell from a representative MD frame. (d) *Z*_F_ ordering of the 19 residues colored by Kyte–Doolittle (KD) class. (e) Spearman correlations between *Z*_F_ and hydration descriptors; hatching indicates descriptors contributing to coordinate construction.

Several diagnostics support *Z*_F_ as the dominant axis of variation. Importantly, the correlation between *Z*_F_ and size-related descriptors does not imply that *Z*_F_ is a size coordinate. Size descriptors were not used as the sole organizing principle, and the fused representation integrates electrostatic, topological, and hydration-level information that cannot be reduced to a molecular dimension alone. No eigengap of the fused random-walk Laplacian exceeds the 0.10 decision threshold, so a single leading coordinate was taken (Figure S5). The descriptors most strongly coupled to *Z*_F_ are the solvation and hydration shell terms of feature set C, one of which also enters the construction of the coordinate and is marked accordingly (Figures 2e and S6). The ordering is robust to stochastic and structural perturbation; leave-one-out refits retain a median Spearman correlation of 0.895 with the full-set coordinate, with Tyr being the largest single drop (0.796) (Figure S7). Redundancy between the feature set similarities is modest, so no pair dominates the fusion (Figure S8). *Z*_F_ therefore reflects a reproducible mode, rather than a fitting artifact.

Plotting residues by their Z_F_ coordinates gives a clear one-dimensional ordering (Figure 2d). Low-polarity, nonpolar-dominated residues occupied one end of the coordinate and highly polar or charge-segmented residues the other, with mixed-character residues falling between. Condition-dependent variations, such as concentration or thermodynamic state, shifted residues along the same axis rather than introducing separate dimensions.

### A Shared Amino Acid Coordinate Extends across Representative Water-Mediated Ordering Processes

Gas hydrates were used as the first application case. Residues within a single library can exhibit promotion-like or inhibition-like behavior, depending on the balance among hydrophobic exposure, electrostatic disruption, interfacial water structuring, and formation kinetics. Along *Z*_F_, the residues fall into four qualitative, chemically coherent regions (Figure 3c), described here as promotion-like, transitional, π/sulfur-motif, and inhibition-like; this grouping is domain-informed rather than derived from a separate clustering. *Z*_F_ itself remains continuous, and residues lie along smooth gradients within and between regions. Moving from low to high *Z*_F_: Leu and Ile anchor the promotion-like region, consistent with hydrophobic interfacial promotion of cage formation. Phe, Met, Trp, and Cys form the adjacent π/surface-active motif, where polarizability and π-surface character contribute to interactions beyond aliphatic hydrophobicity. Val, Thr, Gln, and Pro lie in the transitional region of mixed character, while Asp, Asn, Glu, Lys, and Arg lie at the inhibition-like end, where localized polarity and asymmetric hydration disrupt interfacial water structuring.

**Figure 3:**
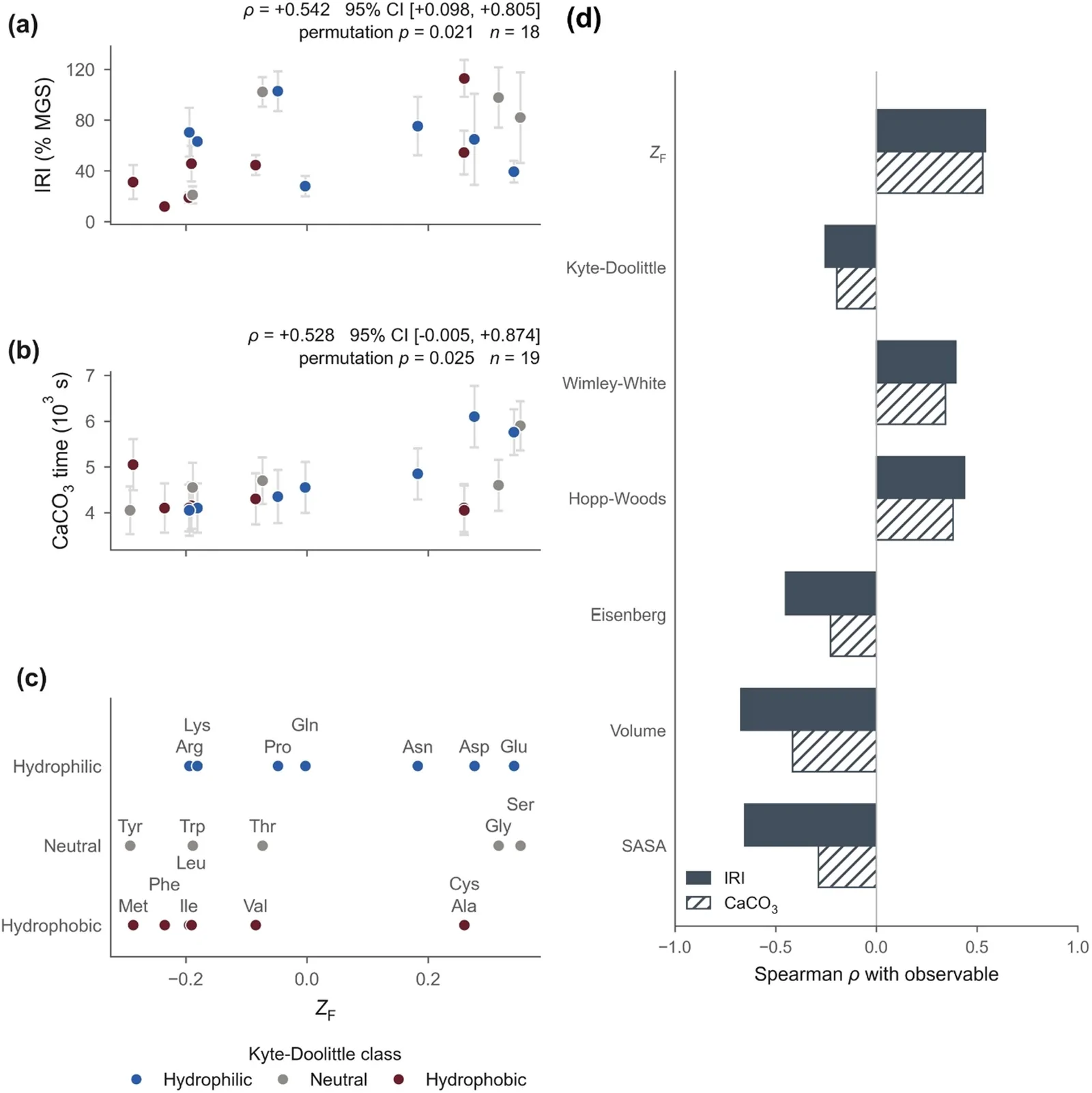
Validation of *Z*_F_ across independent water-mediated processes. (a, b) Correlation of *Z*_F_ with ice recrystallization inhibition and CaCO_3_ nucleation time, respectively, including Spearman ρ, 95% confidence intervals, and permutation p values. (c) Qualitative projection of hydrate-related residue classes onto *Z*_F_. (d) Comparison of *Z*_F_ and established physicochemical scales.

IRI provides a second comparison: it probes water ordering through ice-surface recognition and grain-boundary inhibition during recrystallization. Activity is reported as the mean grain size percentage (% MGS), with lower values indicating stronger inhibition. Amino acids as low-molecular-weight ice recrystallization inhibitors and descriptors that incorporate three-dimensional structure and hydration information improve the estimation of their activity.^2^ Projecting the IRI data onto *Z*_F_ gave ρ*Z*_F_ = +0.542 (*n* = 18; l-Tyr is absent from the data set), opposite in sign to the KD correlation ρ_KD_ = −0.254 (Figure 3a).^4^ *Z*_F_ placed stronger inhibitors (lower % MGS) at the low-*Z*_F_ end and weaker polar or charged residues at the high end; the permutation test rejects the null at the 5% level (p = 0.021), with the absolute correlation strengths comparable at this sample size (Figure 3a,d). The two scales differ in sign rather than magnitude: residues with similar KD values map to different positions along *Z*_F_ and show different % MGS, indicating that *Z*_F_ resolves variation that hydropathy compresses to a single value. Because molecular size and solvent-accessible surface area (SASA) are partially coupled with the learned coordinate in a small amino acid data set, the contribution of size-related effects was further examined (Table 1). Controlling for SASA weakened the association between Z_F_ and both observables, indicating that the observed ordering is not explained by size alone.

**Table 1:** Statistical Validation and Robustness Analysis of the *Z*_F_ Coordinate^a^

	value	95% CI/s	note
primary observables			
IRI, % mean grain size (*n* = 18)	+0.542	[+0.098, +0.805]	permutation *p* = 0.021
CaCO_3_ nucleation time (*n* = 19)	+0.528	[−0.005, +0.874]	permutation *p* = 0.025; CI includes zero
size control (partial ρ, SASA held constant)			
IRI | SASA	+0.062	[−0.548, +0.645]	CI includes zero
CaCO_3_ | SASA	+0.387	[−0.215, +0.816]	CI includes zero
competing loadings on *Z*_F_			
|ρ| with SASA (size)	0.789		difference = 0.005
|ρ| with RDF integral (hydration)	0.784		RDF integral is a construction term
comparator scales (ρ with observable)	IRI	CaCO_3_	
*Z*_F_	+0.542	+0.528	
Kyte–Doolittle	–0.254	–0.196	
Wimley–White	+0.395	+0.343	
Hopp–Woods	+0.437	+0.381	
Eisenberg	–0.451	–0.227	
volume	–0.672	–0.417	
SASA	–0.653	–0.288	
latent dimension			
δ_1_/δ_2_ (production)	0.0625	0.0541	neither clears the preregistered 0.10
stability (ρ with production *Z*_F_)	median	worst	
leave-one-out (19 refits)	0.895	0.796	worst: Tyr removed
*m* = 15 subsample (*B* = 50)	0.827	0.493	
view B seed sweep (20 seeds)	0.930	p5: 0.839	2 of 20 seeds collapsed
view redundancy (signed, preclipping)			
view A – view B	–0.184	–0.124 clipped	clipped value is what SNF consumes

a Spearman correlations of *Z*_F_ with ice recrystallization inhibition and CaCO_3_ nucleation time, with 95% bootstrap confidence intervals, permutation p values, solvent-accessible surface area (SASA)-controlled partial correlations, comparator hydrophobicity/size scales, latent-dimension separations (δ_1_/δ_2_), stability diagnostics, and Feature set A–B redundancy statistics.

CaCO_3_ crystallization provides a mineral-nucleation comparison from aqueous solution. In an earlier systematic screen, Asp was active across multiple stages, Glu and Ser strongly delayed nucleation, and several other residues showed weak or stage-dependent effects.^3^ The contrast between Asp and Glu is informative. Despite differing by only one methylene group, the two residues showed distinct mineralization behavior, indicating that side-chain functionality alone is insufficient. CaCO_3_ nucleation time also tracked Z_F_ with a sign opposite to that of the KD hydropathy scale (ρ*Z*_F_ = +0.528; Figure 3b). The two scales agree on the residue identities of the extremes (Asp, Glu, Ser at one end; aliphatic residues at the other), but place them in opposite directions along the activity axis. The permutation test indicates significance at the 5% level (p = 0.025), whereas the bootstrap interval includes zero at n = 19. Thus, the correlation magnitude should be interpreted cautiously, although the direction of the trend agrees with the IRI result (Figure 3b,d). Polar or charged residues, particularly Asp, Glu, and Ser, occupied the higher-*Z*_F_ side and showed longer nucleation times, while several hydrophobic residues clustered toward the lower-*Z*_F_ side. Although CaCO_3_ crystallization involves multiple coupled stages and the correlation was therefore incomplete, *Z*_F_ captured the broad tendency of polar and charge-segmented residues to perturb early mineralization more effectively than hydropathy alone.

Across the three systems, residue placements along *Z*_F_ reflect a common side-chain chemistry. Aliphatic side chains reduce first-shell water entropy without providing positionally defined hydrogen-bond donors or acceptors; this places Leu and Ile at the low-*Z*_F_ end and supports clathrate-like cage formation in hydrates. Carboxylate and short hydroxyl groups impose the opposite constraint, enforcing localized hydrogen-bond geometries on neighboring waters and binding Ca^2+^ or docking at ice-grain boundaries with positional specificity. Asp, Glu, and Ser therefore fall at the high-*Z*_F_ end for both hydrate inhibition and CaCO_3_ nucleation delay. Val, Thr, Pro, Gly, and Gln combine both characters and sit between these limits, with system-dependent behavior. Phe, Trp, Met, and Cys form a separate group, where polarizability and π-surface contact introduce a third interaction mode beyond aliphatic hydrophobicity.

## Conclusion

Amino acid effects in water-mediated processes are difficult to rationalize within a single application. The same residue can promote hydrate formation under one condition and inhibit it under another, and the IRI and CaCO_3_ crystallization show similar ambiguities. *Z*_F_ condenses the coupled balance of electrostatics, geometry, surface exposure, and hydration into a single coordinate derived from the fused multiview landscape.

The advantage of *Z*_F_ over a one-dimensional hydropathy scale lies in the direction of the induced ordering, not in the magnitude of the rank correlation. In IRI and CaCO_3_ crystallization, *Z*_F_ orders the observable in the opposite direction to KD, placing Asp, Glu, and Ser toward the inhibition-like end of both processes. The reversed sign reflects the underlying chemistry: in IRI, localized charge patches engage ice-grain boundaries through hydrogen-bond donor and acceptor motifs that aliphatic side chains cannot supply. In CaCO_3_ nucleation, carboxylate and hydroxyl groups bind Ca^2+^ and disrupt prenucleation clusters through charge localization rather than through free-energy transfer from a nonpolar phase. The three systems span host–guest framework formation, ice-grain growth, and ionic prenucleation, yet all depend on how amino acids perturb local water structure while balancing polar and nonpolar interactions.

The position along *Z*_F_ clarifies why functional labels depend on the process measured. Residues at the nonpolar low-*Z*_F_ end (Leu, Ile) and at the polar, charge-segmented high-*Z*_F_ end (Asp, Glu, Ser) show consistent behavior across systems, whereas mixed-character residues (Val, Thr, Pro, Gly, and Gln) shift with the target process. A continuous coordinate captures this gradient more naturally than discrete labels such as promoter, inhibitor, ice growth suppressor, or mineralization modifier.

Several limitations apply. With 19 canonical residues, individual rank correlations have wide confidence intervals, and cross-application alignment is supported by the consistent direction of the rank correlations rather than by the magnitude of ρ. Because the present data set consists of small amino acids, molecular size and solvent-accessible surface area are partially coupled with the learned coordinate. Nevertheless, *Z*_F_ is not a size descriptor: size-controlled analyses reduce this association, while the fused coordinate retains contributions from electronic, structural, and hydration-level representations. This framework therefore becomes increasingly informative for larger and more structurally diverse molecules, where size, hydration, and interaction features become less intrinsically coupled.

*Z*_F_ identifies a continuous molecular coordinate that captures interaction-dependent variation not represented by conventional hydropathy scales, links residue position to interpretable side-chain chemistry, and replaces discrete functional labels with a continuous gradient. The same fusion strategy, when extended to noncanonical residues, short peptides, or interface-specific solvation feature sets, offers a route toward molecular coordinates tailored to other modulation areas.

## Methods

### Molecular Data Set and Protonation States

All 20 standard amino acids except histidine were analyzed (*n* = 19). Histidine was excluded because its side-chain protonation and tautomeric equilibria near pH 7 do not permit a single consistent representation. Each residue was modeled in its dominant protonation state at pH 7, a zwitterionic backbone (NH_3_^+^/COO^–^), deprotonated side chains for Asp and Glu (net charge −1), protonated side chains for Lys and Arg (net charge +1), and neutral forms for the remaining residues. Cysteine was represented as the neutral thiol, whereas the minor thiolate species (Cym) was treated separately and excluded from the 19-residue data set. Protonation states and corresponding p*K*_a_ values are listed in the Supporting Information (Note S1 and Table S1).

### Three Complementary Molecular Views

Three complementary descriptor sets were constructed for the 19 amino acids. Feature set A describes electronic structure using density functional theory with implicit aqueous solvation and includes surface electrostatic-potential descriptors, electrostatic-patch descriptors, molecular shape descriptors, and solvent-accessible surface descriptors. Feature set B represents molecular geometry using an E(3)-equivariant graph neural network (EGNN)^11^ trained by self-supervised contrastive learning on DFT-optimized structures. Feature set C describes hydration structure using hand-crafted descriptors derived from explicit solvent molecular dynamics trajectories based on a proximal (closest-atom) radial distribution function.^12,13^ Details of the quantum-chemical calculations, neural network architecture, molecular dynamics simulations, and descriptor definitions are provided in the Supporting Information (Notes S2–S5).

### Similarity Network Fusion

Each descriptor set was converted into a residue–residue similarity network using cosine similarity, followed by construction of a symmetric seven-nearest-neighbor affinity matrix. The four resulting affinity matrices (Feature sets A and B, and the mean and standard-deviation blocks of Feature set C) were integrated using similarity network fusion (SNF).^6^ During network diffusion, a fixed sparse affinity matrix was used for each view,

1 $${P_{v}}^{t+1}=S_{v}\frac{1}{m-1}\sum_{u\neq v}{P_{u}}^{\left(t\right)}{S_{v}}^{T}$$

where *m* is the number of views. The fused network was calculated as the weighted average of the converged affinity matrices using weights of 1.0, 1.0, 0.85, and 0.65 for Feature sets A, B, C-mean, and C-standard deviation, respectively. Additional details of preprocessing, affinity construction, and diffusion are provided in the Supporting Information (Note S6).

### Spectral Coordinate

Spectral embedding was performed on the fused network using the random-walk graph Laplacian,

2 $$L_{\mathrm{rw}}=I-D^{-1}W_{\mathrm{fused}}$$

where *D* is the degree matrix and *W*_fused_ is the fused affinity matrix. The latent dimensionality was determined using an eigengap criterion^14^ with a relative eigengap threshold of 0.10, resulting in a one-dimensional embedding. The resulting coordinate (*Z*_F_) corresponds to the Fiedler vector (the first nontrivial eigenvector), with its sign fixed according to the polar surface fraction as described in the Supporting Information (Note S6).

### Validation and Statistical Analysis

The spectral coordinate was compared to two quantitative water-mediated properties: ice recrystallization inhibition (*n* = 18) and calcium carbonate nucleation time (*n* = 19). Spearman rank correlations were calculated together with 95% bootstrap confidence intervals and two-sided permutation *p* values (10,000 resamples). The results were also compared with the KD hydrophobicity scale,^4^ other hydrophobicity scales, and molecular size descriptors, including molecular volume and solvent-accessible surface area.

To assess the influence of molecular size, partial Spearman correlations were additionally calculated while controlling for the solvent-accessible surface area, molecular weight, and heavy-atom count. The gas hydrate data set was used only for qualitative comparison because it was not obtained under a uniform quantitative protocol. Pipeline robustness was evaluated by a leave-one-out analysis, residue subsampling, and repeated EGNN training with different random seeds. All analyses were performed in Python using NumPy,^15^ SciPy,^16^ scikit-learn,^17^ and PyTorch.^18^ Full computational protocols are provided in the Supporting Information, including the density functional theory calculations, surface electrostatic potential, MEP patch, and molecular shape descriptors,^19–29^ the E(3)-equivariant graph neural network embedding,^30^ the explicit solvent molecular dynamics simulations,^31–36^ and the spectral embedding of the fused similarity network.^37^

## Supplementary Material



## Data Availability

The data and code supporting the findings of this study are available from Zenodo (DOI: 10.5281/zenodo.21464651).^38^ The repository contains the processed multiview descriptor tables, fused similarity network outputs, *Z*_F_ coordinate data, and the code used for descriptor preprocessing, E(3)-equivariant graph neural network embedding, similarity network fusion, spectral embedding, and robustness analyses.

## Notes

Additional figures and tables, residue protonation states, quantum-chemical calculations, surface electrostatic descriptors, molecular shape descriptors, E(3)-equivariant graph neural network embedding, molecular dynamics simulations, hydration descriptors, similarity network fusion and spectral embedding procedures, and coordinate validation analyses (PDF)
