# Supplementary material for: One-Dimensional Molecular Coordinate Orders Amino Acids across Water-Mediated Processes
Source: J Chem Inf Model. 2026 Aug 8;66(16):10119–26. doi: 10.1021/acs.jcim.6c01897 (PMC13508780; doi:10.1021/acs.jcim.6c01897)
Supplement: Supplementary file 1 [file ci6c01897_si_001.pdf]

Supporting Information

A one-dimensional molecular coordinate orders amino acids across water-mediated processes

Yusung Ok[1] and Youngjune Park[1, 2]*

[1] Department of Environment and Energy Engineering, Gwangju Institute of Science and Technology (GIST), 123 Cheomdangwagi-ro, Buk-gu, Gwangju 61005, Republic of Korea

[2] Research Center for Innovative Energy and Carbon Optimized Synthesis for Chemicals (Inn-ECOSysChem), Gwangju Institute of Science and Technology (GIST), 123 Cheomdangwagi-ro, Buk-gu, Gwangju 61005, Republic of Korea

* *Correspondence: young@gist.ac.kr*

Table of Contents

Figure S1. Surface and patch descriptors of Feature set A

Figure S2. Training of the Feature set B encoder

Figure S3. Molecular-dynamics equilibration for all 19 residues

Figure S4. Convergence of the similarity network fusion

Figure S5. Eigengap of the fused Laplacian

Figure S6. Correlations between Z_F and the hydration descriptors

Figure S7. Stability of the one-dimensional coordinate under data perturbation

Figure S8. Redundancy between feature-set similarities

Note S1. Overview of the pipeline and residue set

Table S1. Protonation state assigned to each residue at pH 7

Note S2. Quantum-chemical protocol, surface electrostatics, and MEP-patch construction (Feature set A)

Note S3. Molecular-shape and surface-area descriptors

Note S4. E(3)-equivariant graph-neural-network embedding (Feature set B)

Note S5. Explicit-solvent MD and hand-crafted hydration descriptors (Feature set C)

Note S6. Preprocessing, similarity network fusion, and spectral embedding

Note S7. Coordinate validation

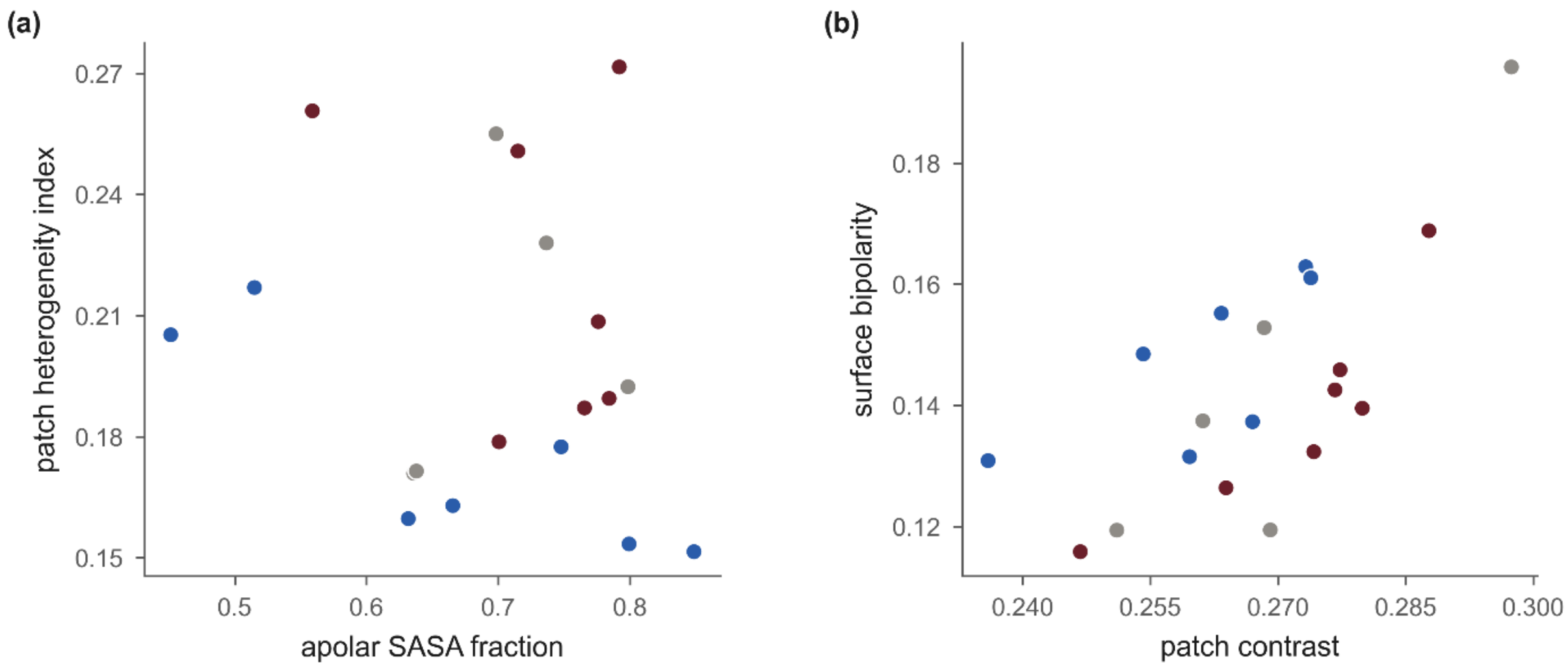

Figure S1. Surface and patch descriptors of Feature set A. Representative pairwise projections of the Feature set A surface descriptors for the 19 residues, colored by Kyte–Doolittle class. (a) Patch heterogeneity index against apolar solvent-accessible surface (SASA) fraction; (b) surface bipolarity against patch contrast. These descriptors are derived from the DFT molecular electrostatic potential evaluated on the ρ = 0.001 a.u. isodensity surface and partitioned into positive and negative charge patches (Note S2). The residues spread broadly in both projections without collapsing onto a single axis, so no individual surface descriptor separates the library on its own, and the electronic view contributes charge- and shape-related information that complements the graph and hydration views.

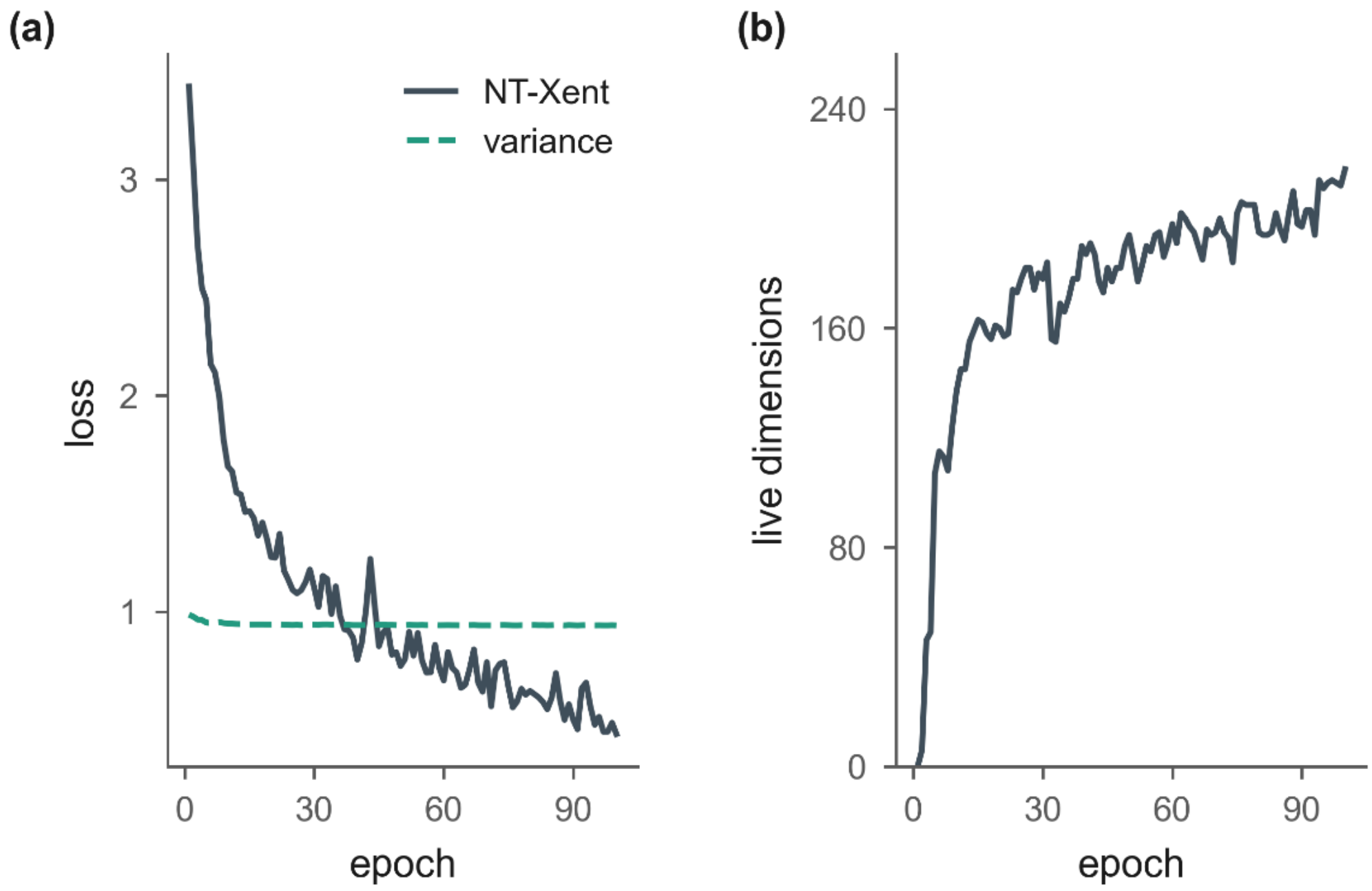

Figure S2. Training of the Feature set B encoder. Diagnostics for the self-supervised contrastive training of the Feature set B E(3)-equivariant graph network (Note S4). (a) NT-Xent contrastive loss and the variance-regularization term over 100 epochs; both decrease smoothly and plateau, indicating stable optimization. (b) Number of embedding dimensions that remain active during training, which stays high and so signals no representational collapse for the reported run.

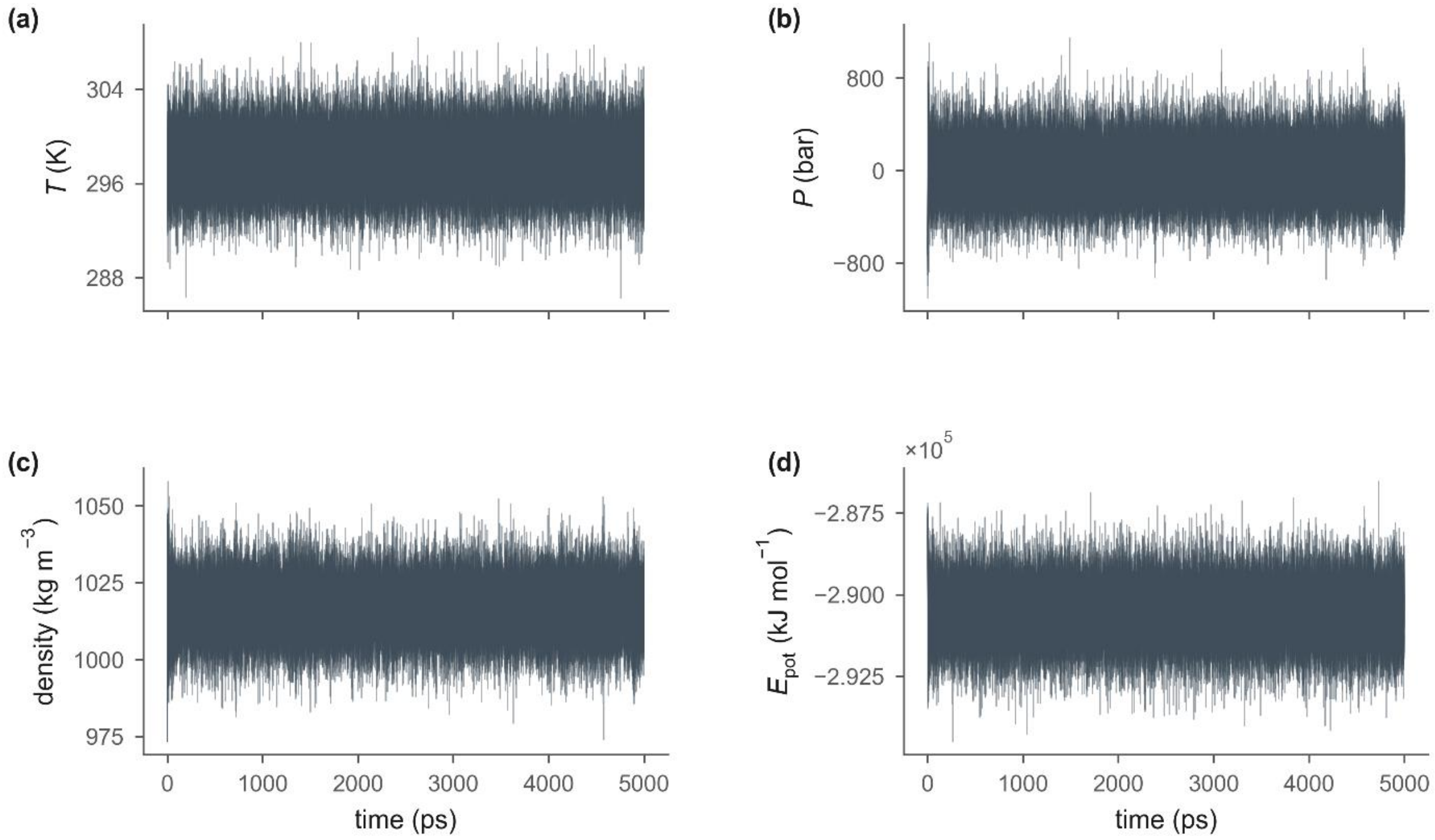

Figure S3. Molecular-dynamics equilibration for all 19 residues. Thermodynamic traces over the production simulations that confirm equilibration of the explicit-solvent systems (Note S5). (a) Temperature, (b) pressure, (c) density, and (d) potential energy against time for the 19 residues, each solvated in CHARMM-modified TIP3P water and simulated with the CHARMM36 force field at 298 K and 1 bar. Every quantity fluctuates around a stable mean with no systematic drift (temperature near 298 K, pressure around 0 bar, density near 1000 kg m^{-3}), confirming the trajectories are equilibrated before the first-shell hydration descriptors are averaged over the final half of each run.

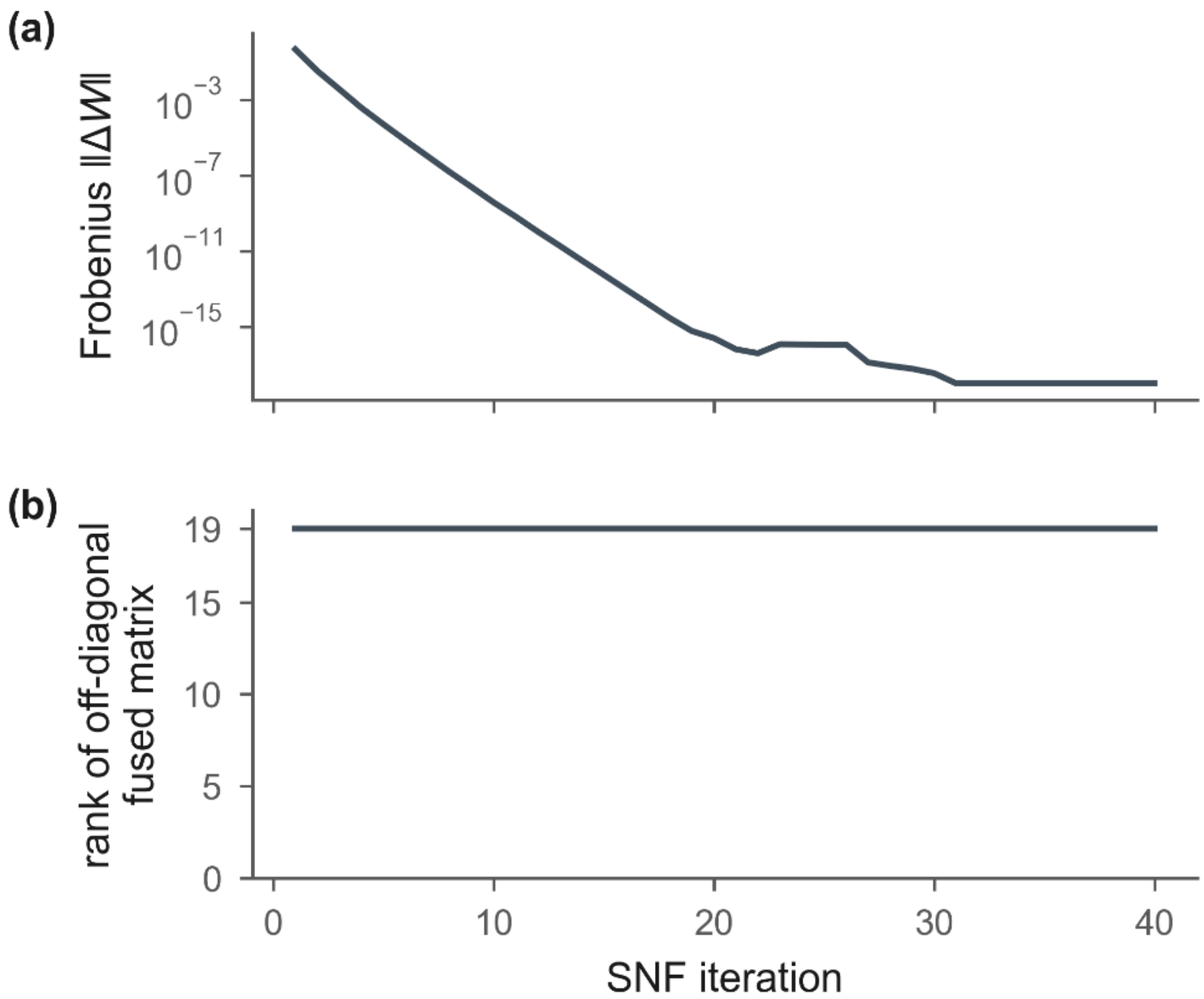

Figure S4. Convergence of the similarity network fusion. Convergence diagnostics for the iterative similarity network fusion (Note S6). (a) Frobenius norm of the change in the fused affinity matrix between successive diffusion iterations; the update decays by roughly fifteen orders of magnitude and reaches machine precision within about thirty iterations, so the fusion converges well before the iteration limit. (b) Rank of the off-diagonal fused matrix, which remains at its full value of 19 throughout, showing that diffusion does not collapse the residues onto a degenerate low-rank structure and that every residue retains a distinct fused profile.

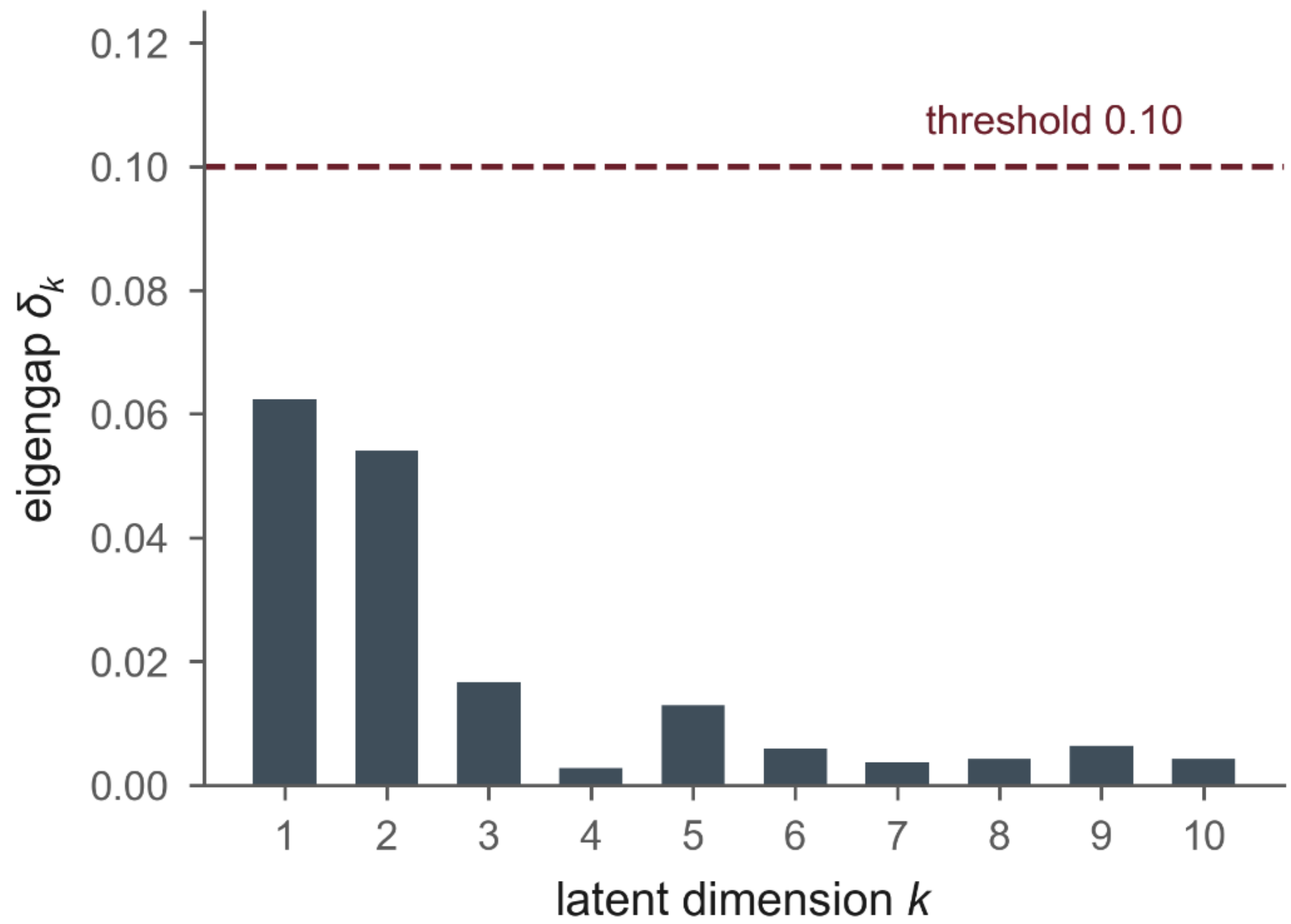

Figure S5. Eigengap of the fused Laplacian. Dimensionality criterion for the spectral embedding (Note S6). The eigengap δ_k is the difference between successive eigenvalues of the fused normalized Laplacian; a gap exceeding the pre-registered threshold of 0.10 would justify a k-dimensional embedding. The two largest gaps ($\delta_1 \approx 0.062$ and $\delta_2 \approx 0.054$) both fall below the threshold and no gap reaches it, so the analysis does not resolve a higher intrinsic dimensionality, and a single leading coordinate (the Fiedler vector, Z_F) is taken as the default rather than as a confirmed multidimensional structure.

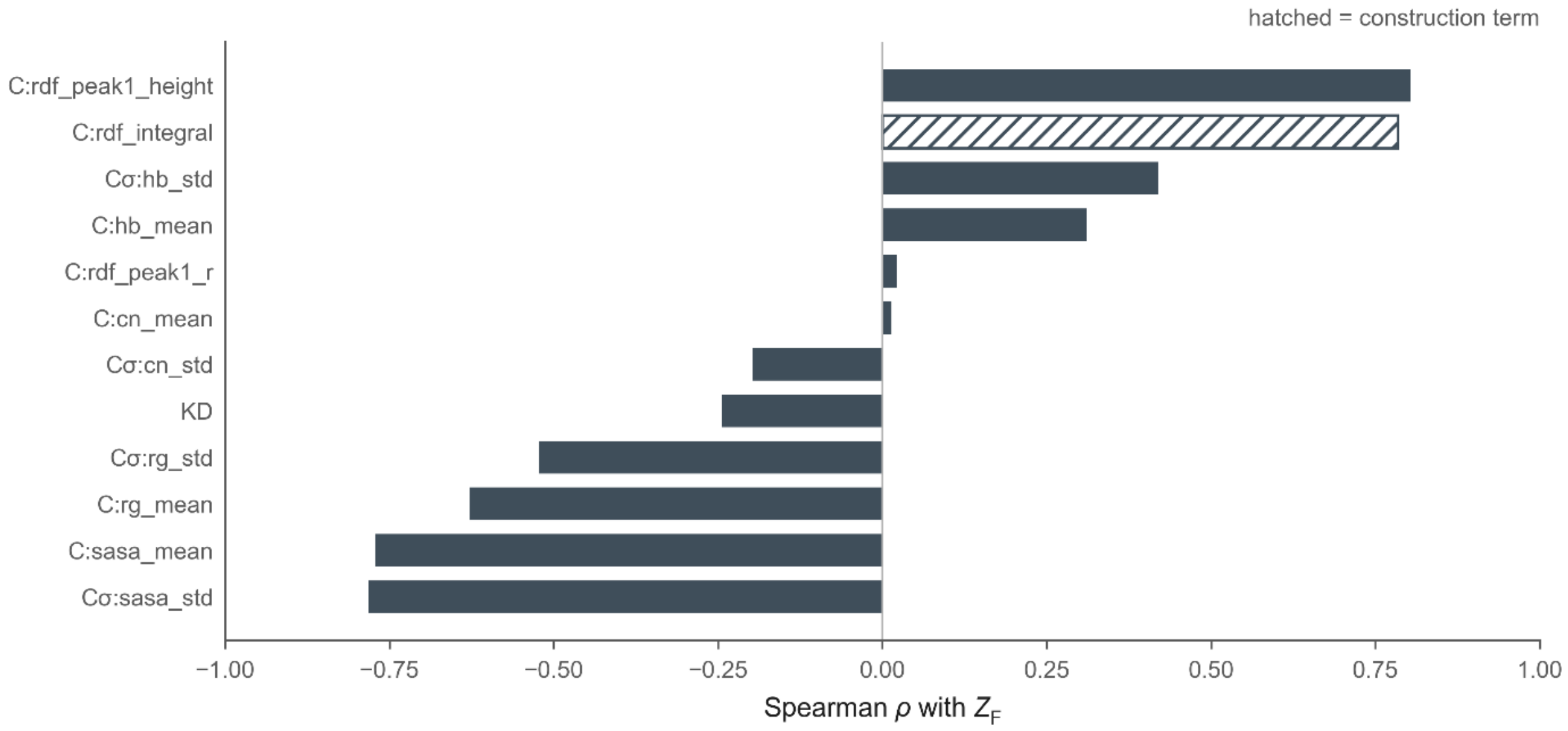

Figure S6. Correlations between Z_F and the hydration descriptors. Spearman rank correlations between Z_F and every Feature set C hydration descriptor across the 19 residues, ordered by correlation, with the Kyte–Doolittle (KD) scale added for reference. The largest positive correlations are with the first-shell radial-distribution peak height and integral, and the strongest negative correlations are with the SASA and radius-of-gyration terms that scale with molecular size. Hatching marks the first-shell RDF integral, which also enters the construction of Z_F and is therefore not an independent correlate. KD couples only weakly to Z_F, consistent with the two scales carrying different information.

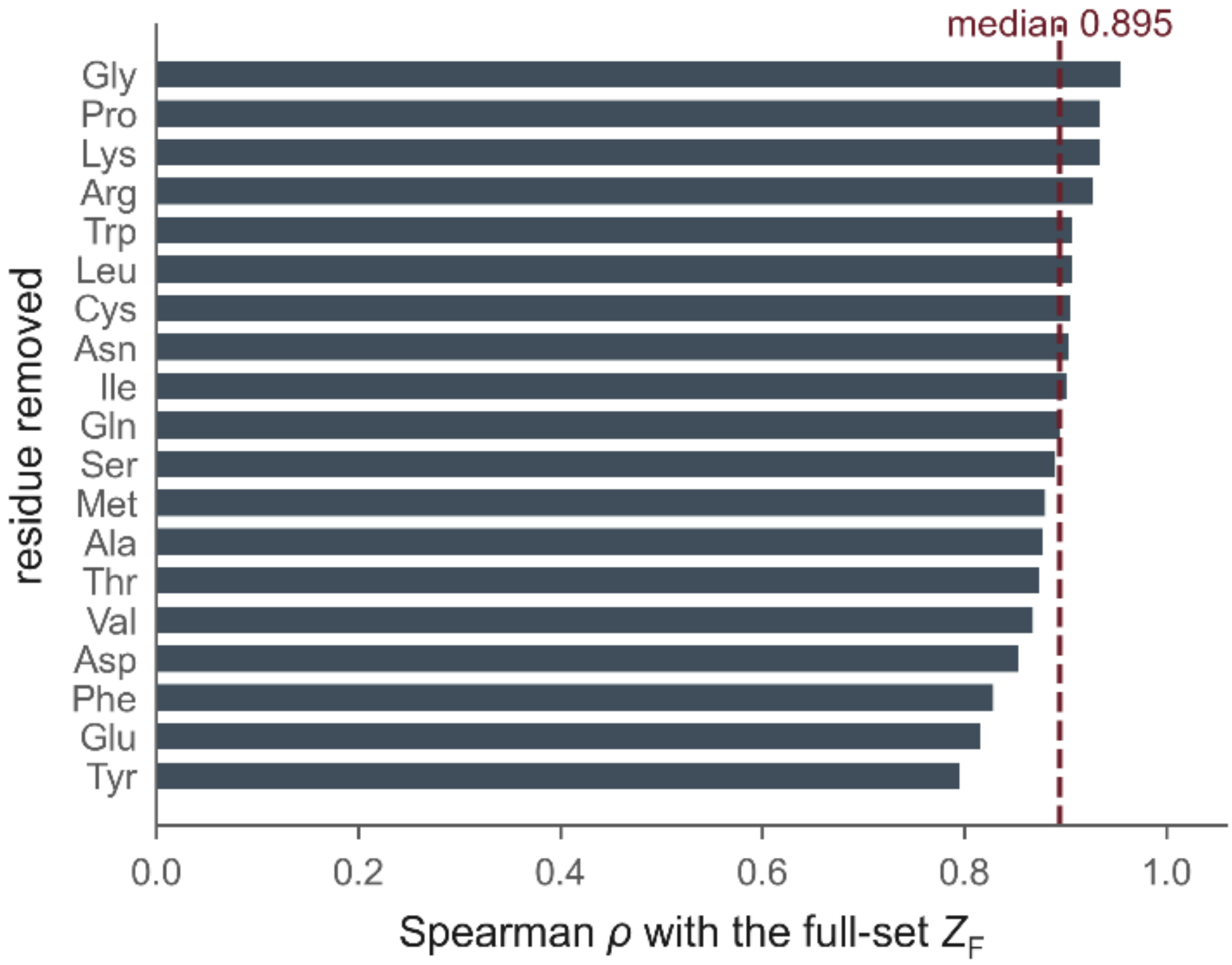

Figure S7. Stability of the one-dimensional coordinate under data perturbation. Stability of the Z_F ordering under resampling and retraining (Note S7). For each residue in turn, the full pipeline from feature generation to spectral embedding is refit with that residue removed, and the resulting coordinate is rank-correlated with the full-set Z_F; the median correlation is 0.895 and the weakest case, removing Tyr, is 0.796, so no single residue drives the axis.

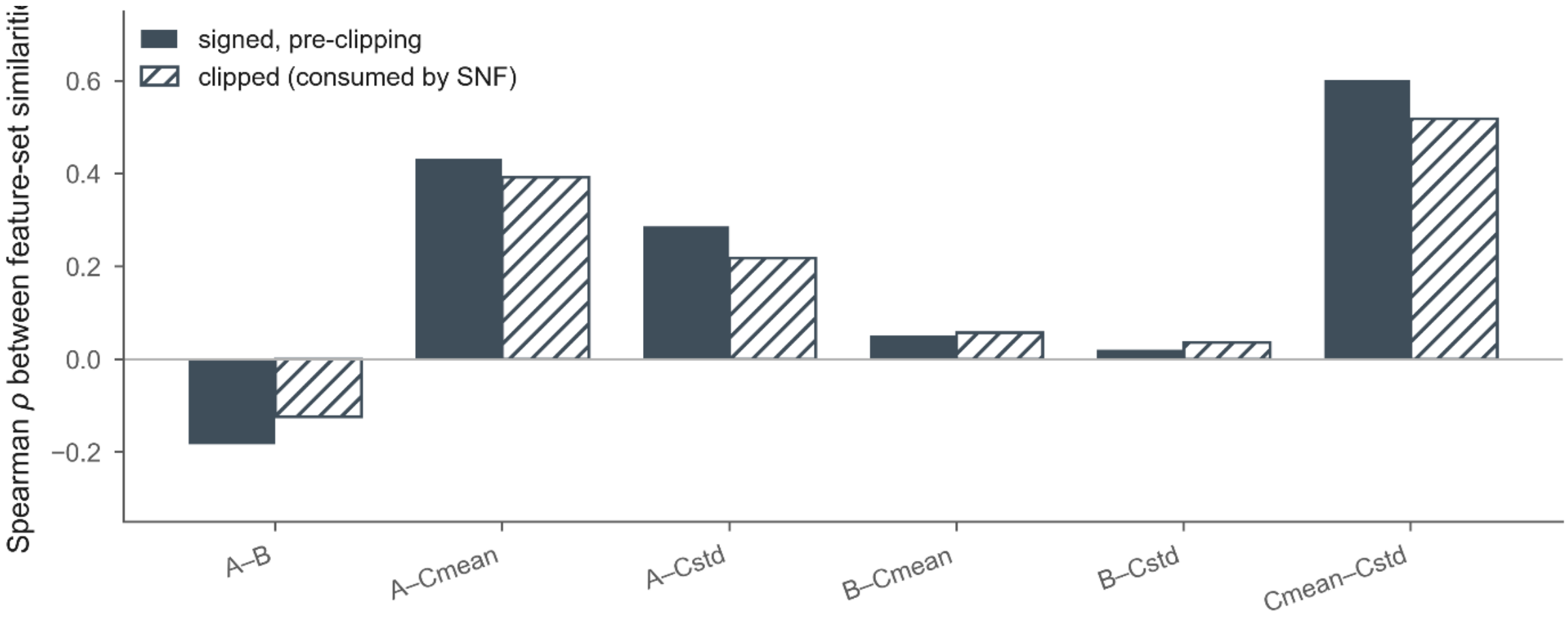

Figure S8. Redundancy between feature-set similarities. Redundancy between the four fused similarity networks, quantified as the Spearman correlation between the pairwise residue similarities of each feature-set pair (Note S6), shown both signed before the non-negativity clipping and as clipped for consumption by the fusion. Most pairs are only weakly correlated, so the feature sets carry largely complementary neighborhood structure; the Feature set A–B pair is slightly negative (−0.184 signed, −0.124 clipped) and the strongest redundancy is between the mean and standard-deviation blocks of Feature set C (C_{mean}–C_{std}), as expected since both derive from the same molecular-dynamics trajectories. The low cross-view redundancy supports fusing the networks rather than treating any pair as interchangeable.

Notes

Note S1. Overview of the pipeline and residue set

Nineteen of the twenty standard amino acids were analyzed, excluding His because its side-chain protonation and tautomeric equilibria near pH 7 preclude a unique representation. Each residue was modeled as its dominant species at pH 7. The backbone was treated in the zwitterionic form (NH_3^+/$COO^−$); Asp and Glu were represented as deprotonated, Lys and Arg as protonated, and all other residues as neutral. Cys was modeled as the neutral thiol, whereas the minor thiolate form (Cym) was analyzed separately and excluded from the 19-residue dataset. This convention ensures that Feature sets A and C describe the same chemical species.

The analysis used three feature sets: Feature set A, density functional theory (DFT)-derived electronic and molecular surface descriptors (Notes S2 and S3); Feature set B, an E(3)-equivariant graph neural network embedding (Note S4); and Feature set C, explicit-solvent molecular dynamics (MD) descriptors of the first hydration shell (Note S5). Feature sets A and B, together with the mean and standard deviation descriptors from Feature set C, were integrated by similarity network fusion, and a single spectral coordinate was extracted (Note S6). Details of the coordinate definition, sign convention, and size-confound analysis are provided in Note S7.

Table S1. Protonation state assigned to each residue at pH 7, the net molecular charge, and the governing ionizable group with a representative pK_a. Backbone α-amino and α-carboxyl groups (representative pK_a ≈ 9.6 and ≈ 2.3) are zwitterionic for all residues and are not repeated per row.

**Residue**	**Dominant species at pH 7**	**Net charge**	**Governing side-chain group (representative pKa)**
Asp	Side-chain carboxylate deprotonated	−1	β-COOH (≈ 3.7)
Glu	Side-chain carboxylate deprotonated	−1	γ-COOH (≈ 4.3)
Lys	Side-chain ammonium protonated	+1	ε-NH3+ (≈ 10.5)
Arg	Side-chain guanidinium protonated	+1	guanidinium (≈ 12.5)
Cys	Neutral thiol (thiolate as Cym sidecar)	0	–SH (≈ 8.3; ≈5% thiolate)
Tyr	Neutral phenol	0	–OH (≈ 10.1)
Ala, Asn, Gln, Gly, Ile, Leu, Met, Phe, Pro, Ser, Thr, Trp, Val	Backbone zwitterion only	0	none ionizable near pH 7

Note S2. Quantum-chemical protocol, surface electrostatics, and MEP-patch construction (Feature set A)

Electronic-structure calculations

Electronic-structure calculations were performed using Gaussian 16 (Revision A.03)[19]. Each residue was modeled in its dominant species at pH 7. Geometry optimization was carried out in aqueous solution at the ωB97X-D/6-311++G(d,p) level[20,21] using the SMD continuum solvation model,[22] with tight SCF convergence and an ultrafine integration grid. Harmonic frequency calculations confirmed that all optimized structures corresponded to local minima. Single-point calculations were subsequently performed at the ωB97X-D/aug-cc-pVTZ level[23] with SMD solvation to obtain the electron density and molecular electrostatic potential (MEP), which were exported as volumetric cube files with a grid spacing of 0.08 Å. Atomic charges were calculated using the CM5 scheme[24], with Hirshfeld, ChelpG, Merz–Singh–Kollman, and Gasteiger charges evaluated for comparison. Initial conformers were generated with RDKit using the MMFF94s force field before DFT optimization.[25]

Surface electrostatic-potential descriptors

The MEP was evaluated on the $\rho = 0.001$ a.u. electron-density isosurface after excluding points within the nuclear exclusion radius. Ten Politzer surface electrostatic-potential descriptors[26,27] were calculated: the minimum and maximum surface potentials (Vmin and Vmax), the mean potential, the mean positive and negative potentials, the positive, negative, and total variances, the average deviation (Π), and the electrostatic balance (ν),

$$\Pi = \frac{1}{n}\sum_i |V_i - \bar{V}| \qquad \text{(S1)}$$

$$\nu = \frac{\sigma^2{}_+\sigma^2{}_-}{(\sigma_{tot})^2}, \quad {\sigma_{tot}}^2 = \sigma^2{}_+ + \sigma^2{}_- \tag{S2}$$

For the charged residues (Asp, Glu, Lys, and Arg), the monopole contribution was removed before descriptor calculation,

$$V'(r) = V(r) - \frac{q}{|r - r_0|}, \quad r_0 = \frac{\sum_i Z_i r_i}{\sum_i Z_i} \tag{S3}$$

where q is the formal charge and r_0 is the center of nuclear charge. Alternative definitions of r_0 produced nearly identical residue orderings (Spearman $\rho \geq 0.98$).

MEP-patch descriptors

Eight MEP-patch descriptors were calculated by partitioning the isodensity surface into contiguous regions of positive and negative electrostatic potential. Surface points were clustered using DBSCAN[28] on the scaled feature vector [x/1.5, y/1.5, z/1.5, V] with eps = 1.0 and a minimum cluster size of 20 points. Positive and negative regions were analyzed separately, and clusters containing fewer than 50 points were discarded. The resulting descriptors included the numbers of positive and negative patches, the Gini coefficient of patch areas, patch contrast, potential dispersion, bipolarity, the patch heterogeneity index, and surface coverage. Sensitivity to the isodensity value was evaluated using isovalues of 0.0005, 0.001, and 0.002 a.u.

Solvent-accessible surface descriptors

Solvent-accessible surface descriptors were computed from the DFT-optimized geometries using the Shrake–Rupley algorithm[29] with Fibonacci sampling (512 points per atom), a probe radius of 1.4 Å, and van der Waals radii of H (1.20 Å), C (1.70 Å), N (1.55 Å),

O (1.52 Å), and S (1.80 Å). Surface points not occluded by neighboring atoms were retained and classified as polar (N, O, and S) or apolar (C and H) according to the parent atom.

Surface descriptors were calculated from the retained surface-point counts. The polar surface fraction was defined as

$$f_{polar} = 1 - \frac{N_{apolar}}{N_{total}} \qquad \text{(S4)}$$

where N_{apolar} and N_{total} denote the numbers of apolar and total surface points, respectively. Area-weighted SASA values were also calculated for comparison.

Note S3. Molecular-shape and surface-area descriptors

Feature set A comprises molecular shape and surface descriptors derived from the DFT-optimized geometry. Five size-intensive shape descriptors (asphericity, eccentricity, the normalized principal moment ratios NPR1 and NPR2, and the spherocity index) were calculated with RDKit[29]. These descriptors complement the surface descriptors by characterizing overall molecular geometry.

Two descriptor groups included in the previous version were excluded. Connectivity-based two-dimensional topological descriptors were removed because they are graph-derived quantities and are represented in Feature set B. In addition, size-extensive shape descriptors (radius of gyration and the unnormalized principal moments of inertia) were excluded because they provide information largely redundant with the surface descriptors.

The final Feature set A contains 27 descriptors: 10 Politzer surface electrostatic-potential statistics, 8 MEP-patch descriptors, 5 molecular shape descriptors, and 4 solvent-accessible surface descriptors. Residual correlations with molecular size were evaluated separately at the coordinate level (Note S7).

Note S4. E(3)-equivariant graph-neural-network embedding (Feature set B)

Each residue was represented as a molecular graph in which atoms were treated as nodes with atomic number as the node feature. Edges were assigned between atoms separated by ≤ 5.5 Å, with the interatomic distance used as the edge attribute. Molecular embeddings were generated using a five-layer E(3)-equivariant graph neural network (EGNN)[11] with 128 hidden units per layer. A mean readout over node representations produced a 256-dimensional molecular embedding.

The network was trained by self-supervised contrastive learning[30] using random rotations, Gaussian coordinate noise ($\sigma = 0.02$ Å), and edge dropout (rate = 0.1). The NT-Xent loss (temperature = 0.2) with variance regularization was optimized using Adam (learning rate $= 1 \times 10^{-3}$, weight decay $= 1 \times 10^{-4}$) for 100 epochs. The resulting embeddings were projected by PCA with whitening, retaining at least 95% of the variance, and subsequently standardized by z-score normalization before similarity calculation.

The EGNN was trained on the DFT-optimized geometries used in Feature set A to ensure a consistent molecular representation across feature sets. Because contrastive learning is stochastic, training was repeated with 20 random seeds. The DFT-trained model generated with seed 1000 was used for the production analysis, whereas the remaining runs were used to assess embedding stability.

Note S5. Explicit-solvent MD and hand-crafted hydration descriptors (Feature set C)

MD simulations

Explicit-solvent molecular dynamics simulations were performed using GROMACS.[31] Each amino acid was modeled in its dominant species at pH 7 using the CHARMM36 force field[32] and solvated in CHARMM-modified TIP3P water in a cubic box with at least 1.2 nm of solvent padding. Energy minimization was followed by 200 ps of NVT equilibration at 298 K using the velocity-rescaling thermostat[33] and 500 ps of NPT equilibration at 1 bar using the Parrinello–Rahman barostat.[34] Production simulations employed a 2 fs integration time step with LINCS bond constraints[35], particle-mesh Ewald (PME)[36] electrostatics, and a 1.0 nm real-space cutoff. Hydration descriptors were calculated from the final 50% of each production trajectory.

Hydration descriptors

For each trajectory frame, the first hydration shell was defined by water oxygen atoms within 3.5 Å of any solute atom, retaining up to the 64 nearest water molecules. Solute and solvent hydrogen atoms were excluded, and all coordinates were centered before analysis.

Feature set C consists of hand-crafted descriptors characterizing the first hydration shell, including the mean and standard deviation of the hydration-shell radius of gyration, SASA, and solute–water hydrogen-bond count, together with the first-shell coordination number, the position and height of the first peak of the radial distribution function (RDF), and the first-shell RDF integral.

Proximal radial distribution function

The radial distribution function was calculated using the proximal (closest-heavy-atom) convention[12,13]. Each water oxygen was assigned to its nearest solute heavy atom so that distances were measured relative to the molecular surface. The shell volume was estimated by Monte Carlo sampling, yielding a proximal RDF that approaches unity in the bulk region. The first-shell RDF integral was evaluated from 0 to 0.35 nm, and additional cutoffs of 0.30 and 0.40 nm were used for sensitivity analysis.

Note S6. Preprocessing, similarity network fusion, and spectral embedding

Each feature set was preprocessed independently. Descriptor values were clipped to the 1st–99th percentile range, near-constant descriptors (standard deviation < 10^{-8}) were removed, and missing values were replaced by the column mean. Feature set A was standardized by z-score normalization. Feature set B was reduced by PCA with whitening (95% variance retained) and subsequently standardized. The mean and standard-deviation blocks of Feature set C were robust-scaled using the interquartile range, and a constant of 10^{-6} was added to the standard-deviation block before scaling.

For each feature set, pairwise residue similarity was computed using the cosine similarity,

$$W_v(i,j) = max\left(\frac{x_i \cdot x_j}{\|x_i\|\|x_j\|}, 0\right) \quad (S5)$$

where negative similarity values were set to zero. A sparse similarity network was constructed for each feature set by retaining the seven nearest neighbors of each residue, followed by symmetrization and normalization. The four resulting networks were integrated using similarity network fusion (SNF)[6] with weights of 1.0, 1.0, 0.85, and 0.65 for Feature sets A, B, C-mean, and C-standard deviation, respectively. The fused network was then analyzed by spectral graph embedding using the symmetric normalized graph Laplacian[14,37],

$$\mathrm{L_{sym}} = I - D^{-\frac{1}{2}} W D^{-\frac{1}{2}}. \quad (S6)$$

The embedding dimension was determined from the eigengap criterion. The first non-trivial eigenvector (Fiedler vector) was used as the one-dimensional latent coordinate (Z_F). The symmetric normalized Laplacian is used here as a numerically stable, similar-matrix route to the random-walk Laplacian ($L_{rw} = I - D^{-1}W$; Eq. 2, main text): the two share eigenvalues, and the Fiedler vector of L_{sym} is rescaled by $D^{-1/2}$ to give the Fiedler coordinate of L_{rw} that defines Z_F. Its sign was fixed so that positive values correspond to larger polar surface fractions.

Note S7. Coordinate validation

The one-dimensional spectral coordinate (Z_F) was used directly for all subsequent analyses. Associations between Z_F and experimental observables were evaluated using Spearman rank correlation. Ice recrystallization inhibition (IRI) ($n = 18$) and calcium carbonate nucleation time ($n = 19$) were analyzed as quantitative benchmarks, together with representative hydrophobicity scales and molecular size descriptors. Confidence intervals and statistical significance were estimated by percentile bootstrap and permutation testing (10,000 resamples each).

Because molecular size may influence both the descriptors and the experimental observables, additional analyses were performed using partial Spearman correlations while controlling for solvent-accessible surface area (SASA), molecular weight, and heavy-atom count. Partial Spearman correlations were calculated by applying Pearson partial correlation to rank-transformed variables.

To evaluate the robustness of the pipeline, the complete workflow was repeated under multiple perturbation schemes, including leave-one-residue-out (LOO) analysis, random residue subsampling, and independent retraining of the EGNN with different random seeds. In each case, the entire workflow, from feature generation to spectral embedding, was repeated without reusing intermediate results.
